# Effect of High‐Intensity, Intermittent, Short‐Duration Re‐Warming up on Cycling Sprint Performance

**DOI:** 10.2478/hukin-2022-0068

**Published:** 2022-09-08

**Authors:** Yuto Yamashita, Yoshihisa Umemura

**Affiliations:** 1 Laboratory for Exercise Physiology and Biomechanics, Graduate School of Health and Sport Sciences, Chukyo University, 101 Tokodachi, Kaizu-cho, Toyota 470-0393, Japan

**Keywords:** sports, sprinting, half-time, intermittent exercise, heart rate, blood lactate concentration

## Abstract

The aim of this study was to investigate the effects of warming up again during half-time (i.e., re-warm up [RW]) with high-intensity, intermittent, short-duration exercise on cycling sprint performance. Participants (male, n = 10) performed intermittent cycling exercise for 40 min, followed by a 15-min half-time period with either rest only (control trials [CON]) or rest followed by a RW (three intervals of 3 s of maximal-effort cycling and 27 s of rest [HII]), after which participants performed the Cycling Intermittent-Sprint Protocol (CISP) to evaluate their sprint performance (17.0 ± 1.4°C, 44.2 ± 7.0% relative humidity). CISP intervals comprised 10 s rest, 5 s maximal effort cycling, and 105 s active recovery at 50% of the maximum oxygen uptake (VO_2max_) and were repeated 10 times. All participants performed both trial variations in randomized order. Peak power output of 5-s cycling sprints during the CISP were significantly higher in HII trials than those in CON trials (CON: 813 ± 109 W, HII: 836 ± 118 W, p < 0.05). Oxygen uptake, blood lactate concentration, and the rating of perceived exertion at the beginning of the second half after the RW were significantly higher in HII trials than those in CON trials (p < 0.05). These results demonstrate that the RW with intermittent, high-intensity, short-duration exercise improved subsequent cycling sprint performance in a thermoneutral environment and may represent a new useful RW strategy.

## Introduction

In many team sports, such as soccer and rugby, players must alternate between low-intensity and high-intensity bouts of activity. Duration of high-intensity running bouts (≥15 km/h) is typically 2–5 s (Mohr et al., 2003; Spencer et al., 2004), but elite soccer players engage in more high-intensity running and sprinting bouts than moderate players during matches (Mohr et al., 2003). It has also been demonstrated that the amount of sprinting during a match is related to the match outcome (Chmura et al., 2018).

In early second-half play, however, the amount of high-intensity running and sprint performance have been found to decline (Bradley et al., 2009; Lovell et al., 2013a; Mohr et al., 2003), and injury risk has been found to increase (Rahnama et al., 2002). One reason is the lack of physical preparation for the second half; during half-time, athletes rest, which decreases core and muscle temperatures (Hammami et al., 2018; Mohr et al., 2004; Russell et al., 2015a). Mohr et al. (2004) reported that core and muscle temperatures decreased by 1.1°C and 2.0°C, respectively. For every 1°C decrease in muscle temperature, power output is reduced by 3% (Sargeant, 1987). Moreover, declines in core temperature during half-time have been found to be significantly correlated with declines in exercise performance (r = 0.63) (Russell et al., 2015b). Thus, rest during half-time, without physical preparation for the second half, has a negative influence on performance in the early second half.

Warming up again during half-time (rewarm up [RW]) has been found to prevent the decline in high-intensity running and sprint performance (Edholm et al., 2015; Hammami et al., 2018; Lovell et al., 2013b). After a period of rest, it was found that 7 min of jogging (at 70% of the maximum heart rate [HR_max_]) (Edholm et al., 2015; Mohr et al., 2004) or 5 min of agility exercise (Lovell et al., 2013b) in the latter part of half-time was sufficient to maintain running and sprint performance; however, players must attend to various tasks during half-time, such as attending team meetings, changing clothes, and receiving medical treatment, so as little as approximately 3 min may be available for a RW during half-time (Towlson et al., 2013). Accordingly, short-duration RW protocols are required to meet this time constraint.

Previous studies have reported that a short-duration (<3 min) RW improved early second-half intermittent sprint performance (Yanaoka et al., 2018a, 2018b, 2020). A RW during half-time comprising cycling for 3 min at 70% of HR_max_, 3 min at 30% of maximum oxygen uptake (VO_2max_), and 1 min at 90% of VO_2max_, improved intermittent sprint performance by 4.7%, 4.2%, and 5.7%, respectively, compared with rest only during half-time (Yanaoka et al., 2018a, 2018b, 2020). Another study showed that a RW with 3 min of jogging, stretching, and jumping improved sprint and jump performance compared with rest only (Fashioni et al., 2020). These studies indicated that a short-duration RW, which is easy to implement during half-time, improved subsequent exercise performance.

In previous studies, most RWs comprised continuous moderate-intensity exercise (Mohr et al., 2004; Yanaoka et al., 2018a, 2020). To the best of our knowledge, no study has investigated the effect of high-intensity intermittent exercise as a RW. Yanaoka et al. (2020) reported that a 1 min cycling RW at 90% of VO_2max_ enhanced muscle activation and suggested that increased muscular activation before the second half was an important factor. Previous research has also indicated that high-intensity voluntary contraction enhanced subsequent muscle contractile responses, in a phenomenon called post-activation potentiation (PAP) (Blazevich and Babault, 2019; Sale, 2002). For example, it was reported that the intensity of 60–84% of 1RM was effective for inducing PAP (Wilson et al., 2013). In addition, a high-intensity warm-up including sprinting was reported to have potential which increased voluntary neuromuscular activation and improved subsequent exercise performance (Bishop et al., 2003c). Silva et al. (2018) reported that a sprint task at the end of the warm-up improved subsequent sprint performance by 2– 3%. Importantly, the specific characteristics of the warm-up before sprint performance are reported to be more significant than the duration of the warm-up (Van den Tillaar et al., 2019). Moreover, a high-intensity warm-up has positive effects on body temperature and aerobic metabolism compared with a low-intensity warm-up, in addition to enhancement of neuromuscular activation (McGown et al., 2015).

Some studies have investigated the effect of different types of a warm-up before competition on performance (Bishop et al., 2003c; Dingley et al., 2020; Fujii et al., 2018). For example, work-matched intermittent and constant-workload warm-ups similarly improved performance at the end of 2 min of maximal cycling (Fujii et al., 2018), and kayak ergometer performance was greater after a high-intensity intermittent warm-up than a constant-workload warm-up (Bishop et al., 2003c); however, it is currently unclear whether a RW can induce similar beneficial effects on subsequent performance. Short-duration (approximately 1 min) RW methods have not been well documented, and the effects of an intermittent short-duration RW on subsequent performance levels are not clear. Therefore, the purpose of the present study was to investigate the effects of a high-intensity, intermittent, short-duration RW on early second-half sprint performance. We hypothesized that a high-intensity, intermittent, short-duration RW would improve cycling sprint performance.

## Methods

### Participants

The number of participants was determined with G*power (v3.1.9.7; Dusseldorf, Germany) using data from a previous study (Yanaoka et al., 2020) that investigated the effects of a high-intensity cycling RW within a very short time-frame on intermittent sprint performance. With an effect size (ES) of 1.0, α-level of 0.05 and power of 0.8 (Yanaoka et al., 2021), the analysis indicated that 10 participants were required. Ten healthy men (age [mean ± standard deviation (SD)] 21 ± 2 years, body height 1.71 ± 0.05 m, body mass 64.4 ± 6.1 kg, VO_2max_ 50.1 ± 5.7 mL·kg^−1^·min^−1^) who routinely performed intermittent exercise (more than 2 days per week) participated in this study. Participants had no record of cardiovascular conditions and did not smoke cigarettes. Before participating in this study, all participants provided written informed consent. During the study, participants were instructed to maintain their regular lifestyle, nutritional habits and physical activity, and were asked to avoid strenuous activity and abstain from ingesting caffeine and alcohol for 24 h before each experimental trial. The study was approved by the Chukyo University Ethics Committee on Research with Human Subjects (approval number: 2020-35) and performed in accordance with the ethical standards of the Declaration of Helsinki.

### Procedures

Participants visited the laboratory three times. At the first visit, each participant completed a graded exercise test on a cycle ergometer (Fujinraijin, Ohashi Chiso Laboratory, Tokyo, Japan) to determine their VO_2max_. After participants took an adequate rest, they performed a familiarization trial which was composed of five sets of the first-half exercise and the Cycling Intermittent-Sprint Protocol (CISP). At the second visit, each participant completed one of two trials (in randomized order), and at the third visit, the second trial was completed. Both trials simulated a competition match, i.e., two intermittent cycling exercise intervals separated by a half-time period. During the half-time period, participants either rested or performed high-intensity intermittent cycling as a RW exercise. Trials were conducted at the same time of day to minimize circadian rhythm differences and were separated by at least 3 days. Each trial was conducted in a laboratory with temperature and humidity maintained at 17.0 ± 1.4°C and 44.2 ± 7.0%, respectively.

### VO_2max_ measurement

A graded exercise test on a cycle ergometer was used to determine VO_2max_: each participant cycled, with instructions to maintain a cadence of 80 rpm, at 100 W for 3 min as a warm-up, and then to increase the load by 20 W every 2 min until volitional exhaustion. Oxygen uptake (V̇O_2_) was analyzed breath-by-breath with an automatic gas analyzer (AE-310s, Minato Medical Science, Japan) and averaged over 30-s intervals. VO_2max_ was determined when two of the following three criteria were met: 1) V̇O_2_ leveling off 2) the heart rate was more than 90% HR_max_ (= 220 − age), and 3) the respiratory exchange ratio was greater than 1.05.

### Experimental trials

**Figure 1:**
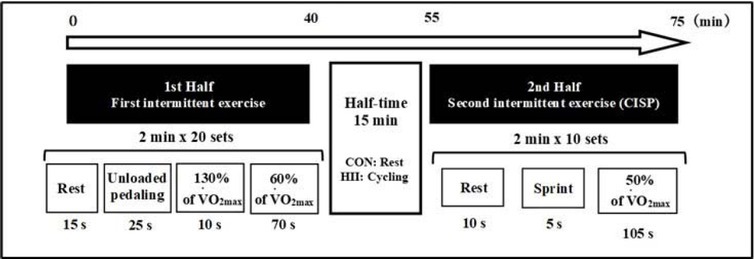
Experimental trial protocol. CISP: Cycling Intermittent-Sprint Protocol; CON: control trials; HII: high-intensity intermittent cycling trials.

The experimental protocol (Figure 1) was based on those of previous studies that investigated the effect of the RW during simulated intermittent team sports matches (Yanaoka et al., 2018a, 2018b, 2020). A cycle ergometer (Fujinraijin, Ohashi Chiso Laboratory, Tokyo, Japan) was used for experimental trials. Participants wore t-shirts and pants. Body weight was measured, and a heart rate monitor (Polar A-300, POLAR, Kempele, Finland) was placed on the chest.

To warm up, participants cycled at 95 W for 5 min, then at 120 W for 30 s, and then rested for 30 s (Yanaoka et al., 2018a). After 5 min of rest while seated on the cycle ergometer, participants performed the first half of the exercise, which comprised 2-min intervals of exercise repeated 20 times. Each interval comprised 15 s rest, 25 s unloaded cycling, 10 s high-intensity cycling (130% of VO_2max_), and then 70 s moderate-intensity cycling (60% of VO_2max_). During the halftime period, participants in the control (CON) trials rested while seated on the ergometer for 15 min, and those in the high-intensity trials (HII) performed high-intensity intermittent short-duration exercise (three intervals comprising 3 s of maximal effort cycling and 27 s of rest, ending 1 min before the start of the second half of exercise) as a RW. On the basis of V̇O_2_, which was similar to that in a previous study (Yanaoka et al., 2020) and the RW of the pilot study, we defined the RW protocol, including the duration of the rest period and the number of sprints. In the second half, the CISP (Hayes et al., 2013) was used to evaluate early second-half performance. The CISP has demonstrated high reliability (Hayes et al., 2013) and comprised ten 2-min intervals (10 s of rest, followed by cycling against resistance of 7.5% of body mass, and then moderate-intensity cycling (50% of VO_2max_) for 105 s at a cadence of 80 rpm).

### Measurements

Power output during 5 s of sprinting of the CISP was captured by the cycle ergometer (every 0.1 s) as performance indicators. Mean power output was calculated every 5 s. To measure respiratory data, participants put on a mask that covered their mouth and nose 5 min before the second half of the exercise began. V̇O_2_, carbon dioxide production (V̇CO_2_), and the respiratory exchange ratio (RER) were recorded continuously from 1 min before the beginning of the second half to the end of the second half, analyzed breath-by-breath (AE-310s, Minato Medical Science, Japan), and averaged every 30 s. The heart rate was continuously recorded every 1 s (Polar A-300, POLAR, Kempele, Finland) and averaged every 30 s. The mean heart rate was calculated at the start of each trial, for 0–40 min, for 54–55 min, and for 55–75 min. Blood lactate concentration (Lactate Pro 2 LT-1730 lactate analyzer, Arkray, Kyoto, Japan) and the rating of perceived exertion (RPE) (Borg, 1982) were measured at the start of each trial and at the 40^th^ min, 55^th^ min, 65^th^ min, and the end of the trial. Because of a technical problem, blood lactate concentration was only captured for nine participants.

### Statistical Analysis

All values are presented as mean ± SD. Peak power output, mean power output, V̇O_2_, V̇CO_2_, RER, mean heart rate, blood lactate concentration, and RPE were compared using two-way (trial and time) repeated measures analysis of variance, and post hoc analyses were performed using Bonferroni correction when a significant interaction was found. Partial η2 was calculated as an estimate of the ES. According to Cohen’s *d*, the ES (Cohen, 1988) was calculated and assessed as small (0.2–0.6), moderate (0.6–1.2), large (1.2–2.0), very large (2.0–4.0), or extremely large (>4.0) (Hopkins et al., 2009). Statistical analysis was conducted using SPSS software (version 26.0, IBM, Armonk, NY, USA). Statistical significance was set at *p* < 0.05.

## Results

### Sprint performance

**Figure 2:**
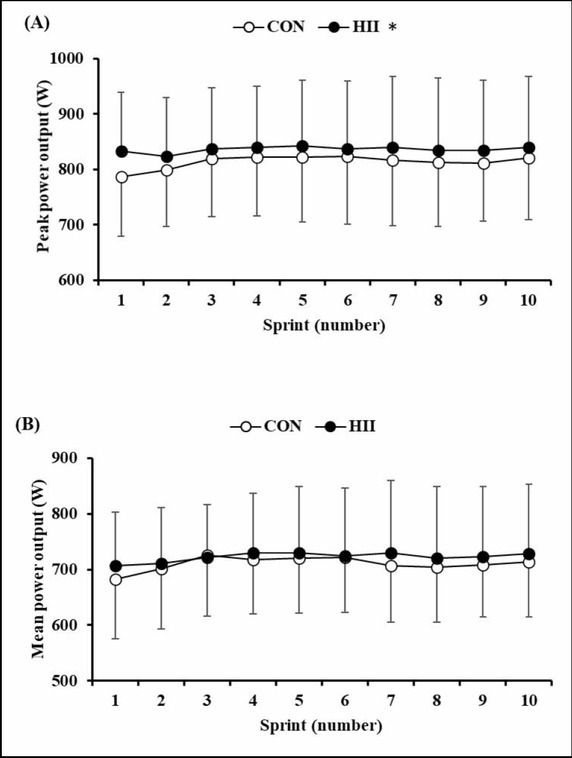
(A) Peak power output and (B) Mean power output during maximal effort cycling of the CISP. CON: control trials; HII: high-intensity intermittent cycling trials. (n = 10, mean ± SD) CISP: Cycling Intermittent Sprint Protocol. * Significant difference between trials (p < 0.05).

There was a significant main effect of the trial (*p* < 0.05, partial η2 = 0.445), but no significant interaction (*p* > 0.05) for peak power output. Peak power output was higher for HII than for CON trials (CON: 813±109 W, HII: 836 ± 118 W, Figure 2). There was no significant main effect of the trial and no interaction for mean power output (CON: 710 ± 99 W, HII: 722 ± 113 W, Figure 2).

### Physiological response

**Figure 3:**
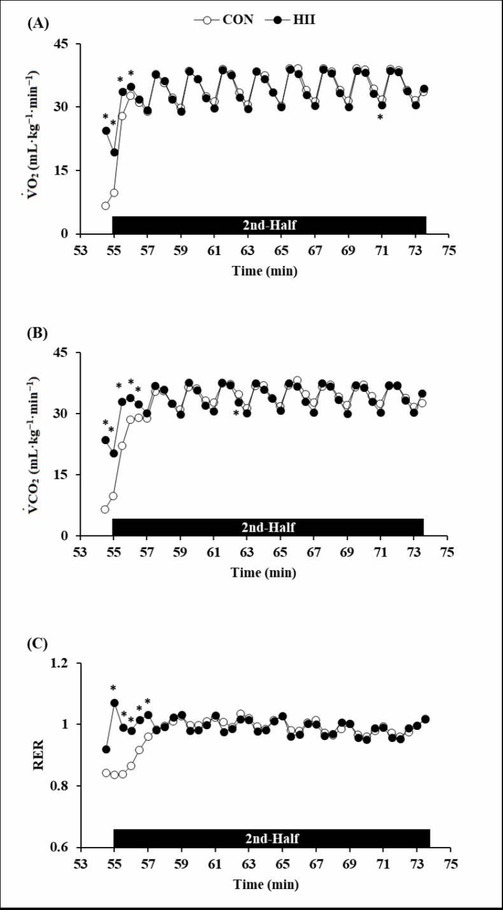
(A) V̇O_2_, (B) V̇CO_2_, and (C) RER from 1 minute before the start of the second half to the end of the second half. CON: control trials; HII: high-intensity intermittent cycling trials. (n = 10, mean ± SD) V̇O_2_: main effect of time; p < 0.05, interaction; p < 0.05. V̇CO_2_: main effect of time; p < 0.05, interaction; p < 0.05. RER: main effect of time; p < 0.05, interaction; p < 0.05. * Significant difference between trials at time points (p < 0.05).

There was a significant interaction for V̇O_2_, V̇CO_2_, RER, heart rate, and blood lactate concentration (V̇O_2_: *p* < 0.05, partial η2 = 0.774, V̇CO_2_: *p* < 0.05, partial η2 = 0.538, RER: *p* < 0.05, partial η2 = 0.519, heart rate: *p* < 0.05, partial η2 = 0.914, blood lactate concentration: *p* < 0.05, partial η2 = 0.369). V̇O_2_ and V̇CO_2_ and RER values were higher in HII trials than those in CON trials in the early part of the second half (V̇O_2_: *p* < 0.05, d = 0.47–6.67: small-extremely large, Figure 3, V̇CO_2_: *p* < 0.05, d = 0.60–3.31: moderate-very large, Figure 3, RER: *p* < 0.05, d = 1.08–2.36: moderate-very large, Figure 3). Mean heart rates from the 54^th^ min to the 55^th^ min were higher in HII trials than those in CON trials (*p* < 0.05, d = 3.08: very large, Table 1). Blood lactate concentration at the 55^th^ min was significantly higher in HII trials than in CON trials (*p* < 0.05, d = 2.00: very large, Table 2).

**Table 1:** Heart rate (HR) throughout trials.

	Time (min)	CON	HII
HR (bpm)	pre	89.1±15.3	86.2±8.4
	1st-Half	130.2±10.7	130.4±9.7
	54–55 min	88.3±13.3	124.1±8.2*
	2nd-Half	149.2±12.4	150.1±10.0

CON: control trials; HII: high-intensity intermittent cycling trials. (n = 10, mean ± SD) * Significant difference between trials (p < 0.05).

**Table 2:** Blood lactate concentration and the rating of perceived exertion (RPE) throughout trials.

	Time (min)	CON	HII
Blood lactate concentration(mmol/L)	pre	1.7±0.4	1.6±0.8
	40	2.2±0.9	2.3±0.8
	55	1.5±0.4	3.7±1.4*
	65	7.1±3.0	7.9±2.9
	post	8.0±4.0	9.1±3.3

RPE	pre	7.7±1.7	8.2±1.8
	40	13.6±1.3	13.7±1.9
	55	8.7±2.2	12.7±2.4^*^
	65	15.7±1.3	15.8±1.2
	post	17.2±1.4	16.9±1.7

CON: control trials; HII: high-intensity intermittent cycling trials (blood lactate concentration: n = 9, mean ± SD RPE: n = 10, mean ± SD). * Significant difference between trials (p < 0.05).

### RPE

There was a significant interaction for the RPE (*p* < 0.05, partial η2 = 0.650). RPE values at the 55^th^ min were higher for HII trials than those for CON trials (*p* < 0.05, d = 1.66: large, Table 2).

## Discussion

We investigated the effects of a high-intensity, intermittent, short-duration RW during half-time on early second-half sprint performance and found that the RW, comprising 3 intervals of 3 s of maximal effort cycling with 27 s of rest, significantly improved peak power output of cycling sprints during the 20-min CISP compared with rest only during half-time.

Although the RW improves early second-half performance (Hammami et al., 2018; Mohr et al., 2004; Yanaoka et al., 2020), RW protocols have not been fully investigated. It can be difficult to implement a RW when there are time constraints; a previous study reported that only approximately 3 min is available for a RW during half-time (Towlson et al., 2013). Therefore, if the duration of the RW can be shorter and still exhibit a beneficial effect, the RW could be adopted as a practical strategy. However, few studies have investigated the effect of a 1-min RW (Yanaoka et al., 2020, 2021). The present study provides new insight regarding the effects of a 1-min RW, which may have beneficial applications for coaches and trainers. We found that 3-s duration of maximal effort in the RW in the present study was equivalent to 20-m sprint time (Gil et al., 2018; Paul et al., 2014). If the present study used free running rather than cycling exercise, it may be possible that performing three sets of a 20 m sprint-RW would induce a similar improvement in subsequent sprint performance. The present study provides a basis for developing practical RW protocols.

In the present study, V̇O_2_ baseline values at the beginning of the second half increased with the RW. In addition, the mean HR and the RPE at the beginning of the second half were higher with the RW (HR: CON, 88.3 ± 13.3 bpm, HII, 124.1 ± 8.2 bpm, RPE: CON, 8.7 ± 2.2, HII, 12.7 ± 2.4). Yanaoka et al. (2020) investigated a 1-min high-intensity (90% of VO_2max_) RW and reported that V̇O_2_ was higher immediately after the beginning of the second half after the RW compared with a half-time with rest only. The RW protocol in the present study involved intermittent and high-intensity exercise. In contrast, Yanaoka et al. (2020) conducted an RW protocol with a constant load of high-intensity exercise. Nevertheless, changes in V̇O_2_ and the mean HR after the RW were similar between the two studies (Yanaoka et al., 2020). A RW during half-time may interfere with player’s psychological preparation (Towlson et al., 2013). In the present study, although participants performed exercise with maximal effort during the RW, RPE values after the RW were relatively low. The V̇O_2_ value, mean HR and RPE after the RW with high-intensity, intermittent, short-duration exercise showed similar values compared with previous studies that simulated intermittent team sports, which also reported beneficial effects of a short-duration RW (Yanaoka et al., 2018a, 2018b, 2020). Thus, the RW protocol in the present study may have elevated V̇O_2_ and the mean HR before the second half, as reported in previous studies, but with less psychological strain.

Mean peak power output for 5 s of maximal effort cycling during CISP intervals was greater by 2.8% in HII trials than in CON trials. In previous studies, when participants performed a RW, performance improved significantly compared with performance after 15 min of passive rest: 3 min of cycling at 70% of HR_max_ as the RW improved mean work in the initial five cycling sprints of the CISP (Yanaoka et al., 2018a); 1 min of cycling at 90% of VO_2max_ improved mean work during cycling sprints in a 10-min CISP (Yanaoka et al., 2020); and moderate-intensity running for 7 min improved 30-m sprint times (Mohr et al., 2004). In the present study, although there were no significant differences in mean power output, we found a significant difference in peak power output. The physiological rationale underlying the difference between peak power output and mean power output in the current results is unclear. However, mean power output of the first sprint and the latter sprints tended to be higher in HII compared with CON trials, although these differences were not significant. We believe that peak power output improved in the present study, because peak power output is a predictor of exercise performance, such as sprinting (Cheetham et al., 1986). The ability to perform high-intensity running and sprinting is an important indicator for team sport players. Thus, improvement in peak power output would have practical implications.

In the present study, regarding peak power output in the first and second sprints, the difference between CON and HII trials was larger than that in other sprints, although the interaction was not significant. The reason for this improvement is unclear because we did not measure muscular activity or metabolism. However, it is possible that this increased performance was related to an increase in neuromuscular activation. The RW in the present study consisted of maximal effort cycling, in which participants were required to perform large voluntary contractions. This may have induced PAP, because it has been reported that PAP can enhance various types of exercise performance, such as jumping and sprinting (McGowan et al., 2015). Another previous study reported greater peak power and average power during kayak ergometer performance after intermittent, high-intensity exercise involving a sprint warm-up, compared with a continuous warm-up (Bishop et al., 2003c). It has been proposed that improved performance might be caused by increased neuromuscular activation (Bishop, 2003b). Moreover, Yanaoka et al. (2020) reported that a cycling RW of 1 min at 90% of VO_2max_ caused enhancement in muscle activation during subsequent intermittent cycling sprints. It is possible that maximal effort cycling during halftime led to PAP, thus improving subsequent intermittent cycling sprint performance. However, cycling sprint performance significantly improved through 10 sprints in the second half of exercise because the RW increased V̇O_2_, indicating that aerobic metabolism was used and anaerobic capacity was conserved for the later use (Bishop, 2003a; McGowan et al., 2015). Furthermore, oxygen supply to the muscle may have been increased by the RW. Phosphocreatine is the main energy source in sprint exercise for resynthesis of adenosine triphosphate, and oxygenation of muscle affects how much phosphocreatine is resynthesized (Hamaoka et al., 1996). Yanaoka et al. (2018) reported that there was a correlation (r = 0.52) between intermittent sprint performance and the mean change in oxygenated hemoglobin concentration during active recovery after sprinting. Thus, HII may increase oxygenation of the muscle after sprinting and promote phosphocreatine resynthesis.

Blood lactate concentrations at the beginning of the second half were also increased by the RW (CON: 1.5 ± 0.4 mmol·L^−1^, HII: 3.7 ± 1.4 mmol·L^−1)^. We propose that a Bohr shift in the O_2_ dissociation curve may have occurred because of a reduction in muscle pH, increasing O_2_ availability. A small reduction in pH may stimulate increased muscle excitability during subsequent exercise bouts (Hansen et al., 2005; Nielsen et al., 2001). Therefore, blood lactate concentrations in the range of 3–5 mmol·L^−1^ are related to better performance (Burnley et al., 2005; Jones et al., 2003; Palmer et al., 2009). The intermittent high-intensity warm-up increased blood lactate concentration (3.2 ± 1.4 mmol·L^−1^) and improved supramaximal kayak ergometer performance (Bishop et al., 2003c). Similarly, in the present study, elevated blood lactate concentrations before exercise may have improved cycling sprint performance in the second half.

The present study had some limitations. First, we simulated the intermittent exercise involved in team sport matches using only one type of exercise (cycling) to evaluate the metabolism mechanisms of the RW. However, intermittent team sports comprise running, physical contact, and other types of exercise. Thus, it is unknown whether this RW protocol will have the same effect on performance during actual matches. However, it has been suggested that there is a moderate correlation between repeated sprint cycling performance and free-running (Fitzsimons et al., 1993). Moreover, previous studies reported similar positive effects on intermittent sprint performance after performing similar RW protocols with cycling (Yanaoka et al., 2020) and running (Yanaoka et al., 2021). Thus, the RW protocol tested in the present study may have the potential to improve intermittent sprint performance during actual matches. Second, the effect of performing the RW in different environments is not clear. Core temperature and muscle temperature decline when the player rests during half-time (Mohr et al., 2004; Russell et al., 2015b), and a correlation (r = 0.60) between decreases in muscle temperature during half-time and decreased sprint performance has been reported (Mohr et al., 2004). In cold environments, core and muscle temperatures may decrease to a greater extent than in thermoneutral environments. In future studies, the effect of environmental conditions on RW protocols should be investigated. Third, we did not measure body temperature and muscular metabolism. Such data may provide important information about physiological responses. Fourth, the present study was not able to clarify whether the RW has the potential to decrease injury risks during the second half, because no data were collected to test this possibility. Finally, there was no specific rationale for the duration of the rest period during the RW. Future studies should determine the effect of the RW on the risk of injury and the optimal high-intensity, intermittent, short-duration RW protocol.

## Conclusions

A high-intensity, intermittent, short-duration RW that can be completed in approximately 1 min during half-time improves second-half cycling sprint performance. These findings suggest that a high-intensity, intermittent, short-duration RW may provide a beneficial half-time strategy.

## References

[j_hukin-2022-0068_ref_001] Bishop D. (2003a). Warm up I: potential mechanisms and the effects of passive warm up on exercise performance. Sports Medicine.

[j_hukin-2022-0068_ref_002] Bishop D. (2003b). Warm up II: performance changes following active warm up and how to structure the warm up. Sports Medicine.

[j_hukin-2022-0068_ref_003] Bishop D., Bonetti D., Spencer M. (2003c). The effect of an intermittent, high-intensity warm-up on supramaximal kayak ergometer performance. Journal of Sports Sciences.

[j_hukin-2022-0068_ref_004] Blazevich A. J., Babault N. (2019). Post-activation potentiation versus post-activation performance enhancement in humans: historical perspective, underlying mechanisms, and current issues. Frontiers in Physiology.

[j_hukin-2022-0068_ref_005] Borg G. A. V. (1982). Psychophysical bases of perceived exertion. Medicine and Science in Sports and Exercise.

[j_hukin-2022-0068_ref_006] Bradley PS., Sheldon W., Wooster B., Olsen P., Boanas P., Krustrup P. (2009). High-intensity running in English FA Premier League soccer matches. Journal of Sports Sciences.

[j_hukin-2022-0068_ref_007] Burnley M., Doust J. H., Jones A. M. (2005). Effects of prior warm-up regime on severe-intensity cycling performance. Medicine and Science in Sports and Exercise.

[j_hukin-2022-0068_ref_008] Cheetham ME, Boobis LH, Brooks S, Williams C. (1986). Human muscle metabolism during sprint running. Journal of Applied Physiology.

[j_hukin-2022-0068_ref_009] Chmura P., Konefał M., Chmura J., Kowalczuk E., Zajac T., Rokita A., Andrzejewski M. (2018). Match outcome and running performance in different intensity ranges among elite soccer players. Biology of Sport.

[j_hukin-2022-0068_ref_010] Cohen J. (1988). Statistical power analysis for the behavioral sciences.

[j_hukin-2022-0068_ref_011] Dingley A. F., Willmott A. P., Fernandes J. F. T. (2020). Self-Selected Versus Standardised Warm-Ups; Physiological Response on 500 m Sprint Kayak Performance. Sports.

[j_hukin-2022-0068_ref_012] Edholm P., Krustrup P., Randers M. B. (2015). Half-time re-warm-up increases performance capacity in male elite soccer players. Scandinavian Journal of Medicine and Science in Sports.

[j_hukin-2022-0068_ref_013] Fashioni E., Langley B., Page R. M. (2020). The effectiveness of a practical half-time re-warm-up strategy on performance and the physical response to soccer-specific activity. Journal of Sports Sciences.

[j_hukin-2022-0068_ref_014] Fitzsimons M., Dawson B., Ward D., Wilkinson A. (1993). Cycling and running tests of repeated sprint ability. Australian Journal of Science and Medicine in Sport.

[j_hukin-2022-0068_ref_015] Fujii N., Hara H., Enomoto Y., Tanigawa S., Nishiyasu T. (2018). Effects of work-matched supramaximal intermittent vs. submaximal constant-workload warm-up on all-out effort power output at the end of 2 min of maximal cycling. European Journal of Sport Science.

[j_hukin-2022-0068_ref_016] Gil S., Barroso R., Crivoi do Carmo E., Loturco I., Kobal R., Tricoli V., Ugrinowitsch C., Roschel H. (2018). Effects of resisted sprint training on sprinting ability and change of direction speed in professional soccer players. Journal of Sports Sciences.

[j_hukin-2022-0068_ref_017] Hamaoka T., Iwane H., Shimomitsu T., Katsumura T., Murase N., Nishio S., Osada T., Kurosawa Y., Chance B. (1996). Noninvasive measures of oxidative metabolism on working human muscles by near-infrared spectroscopy. Journal of Applied Physiology.

[j_hukin-2022-0068_ref_018] Hammami A., Zois J., Slimani M., Russel M., Bouhlel E. (2018). The efficacy and characteristics of warm-up and re-warm-up practices in soccer players: A systematic review. Journal of Sports Medicine and Physical Fitness.

[j_hukin-2022-0068_ref_019] Hansen A. K., Clausen T., Nielsen O. B. (2005). Effects of lactic acid and cat- echolamines on contractility in fast-twitch muscles exposed to hyperkale- mia. American Journal of Physiology-Cell Physiology.

[j_hukin-2022-0068_ref_020] Hayes M., Smith D., Castle P. C., Watt P. W., Ross E. Z., Maxwell N. S. (2013). Peak power output provides the most reliable measure of performance in prolonged intermittent-sprint cycling. Journal of Sports Sciences.

[j_hukin-2022-0068_ref_021] Hopkins W. G., Marshall S. W., Batterham A. M., Hanin J. (2009). Progressive statistics for studies in sports medicine and exercise science. Medicine and Science in Sports and Exercise.

[j_hukin-2022-0068_ref_022] Jones A. M., Wilkerson D. P., Burnley M., Koppo K. (2003). Prior Heavy Exercise Enhances Performance during Subsequent Perimaximal Exercise. Medicine and Science in Sports and Exercise.

[j_hukin-2022-0068_ref_023] Lovell R., Barrett S., Portas M., Weston M. (2013a). Re-examination of the post half-time reduction in soccer work-rate. Journal of Science and Medicine in Sport.

[j_hukin-2022-0068_ref_024] Lovell R., Midegley A., Barret S., Carter D., Small K. (2013b). Effect of different half-time strategies on second half soccer-specific speed, power and dynamic strength. Scandinavian Journal of Medicine and Science in Sports.

[j_hukin-2022-0068_ref_025] McGowan C. J., Pyne D. B., Thompson K. G., Rattray B. (2015). Warm-Up Strategies for Sport and Exercise: Mechanisms and Applications. Sports Medicine.

[j_hukin-2022-0068_ref_026] Mohr M., Krustrup P., Nybo L., Nielsen J. J., Bangsbo J. (2004). Muscle temperature and sprint performance during soccer matches - Beneficial effect of re-warm-up at half-time. Scandinavian Journal of Medicine and Science in Sports.

[j_hukin-2022-0068_ref_027] Mohr M., Krustrup P., Bangsbo J. (2003). Match performance of high-standard soccer players with special reference to development of fatigue. Journal of Sports Sciences.

[j_hukin-2022-0068_ref_028] Nielsen O. B., de Paoli F., Overgaard K. (2001). Protective effects of lactic acid on force production in rat skeletal muscle. Journal of Physiology.

[j_hukin-2022-0068_ref_029] Palmer C. D., Jones A. M., Kennedy G. J., Cotter J. D. (2009). Effects of prior heavy exercise on energy supply and 4000-m cycling performance. Medicine and Science in Sports and Exercise.

[j_hukin-2022-0068_ref_030] Paul C., Stewart A., Bloom L., Ben C. (2014). Relationships between strength, sprint, and jump performance in well-trained youth soccer players. Journal of Strength and Conditioning Research.

[j_hukin-2022-0068_ref_031] Rahnama N., Reilly T., Lees A. (2002). Injury risk associated with playing actions during competitive soccer. British Journal of Sports Medicine.

[j_hukin-2022-0068_ref_032] Russell M., West D. J., Harper L. D., Cook C. J., Kilduff L. P. (2015a). Half-Time Strategies to Enhance Second-Half Performance in Team-Sports Players: A Review and Recommendations. Sports Medicine.

[j_hukin-2022-0068_ref_033] Russell M., West D. J., Briggs M. A., Bracken R. M., Cook C. J., Giroud T., Gill N., Kilduff L. P. (2015b). A passive heat maintenance strategy implemented during a simulated half-time improves lower body power output and repeated sprint ability in professional Rugby Union players. PLOS ONE.

[j_hukin-2022-0068_ref_034] Sale D. G. (2002). Postactivation potentiation: role in human performance. Exercise and Sport Sciences Reviews.

[j_hukin-2022-0068_ref_035] Sargeant A. J. (1987). Effect of muscle temperature on leg extension force and short-term power output in humans. European Journal of Applied Physiology Occupational Physiology.

[j_hukin-2022-0068_ref_036] Silva L. M., Neiva H. P., Marques M. C., Izquierdo M., Marinho D. A. (2018). Effects of warm-up, post-warm-up, and re-warm-up strategies on explosive efforts in team sports: a systematic review. Sports Medicine.

[j_hukin-2022-0068_ref_037] Spencer M., Lawrence S., Rechichi C., Bishop D., Dawson B., Goodman C. (2004). Time-motion analysis of elite field hockey, with special reference to repeated-sprint activity. Journal of Sports Sciences.

[j_hukin-2022-0068_ref_038] Towlson C., Midgley A. W., Lovell R. (2013). Warm-up strategies of professional soccer players: Practitioners’ perspectives. Journal of Sports Sciences.

[j_hukin-2022-0068_ref_039] Van den Tillaar R, Lerberg E, vonHeimburg E. (2019). Comparison of three types of warm-up upon sprint ability in experienced soccer players. Journal of Sports and Health Science.

[j_hukin-2022-0068_ref_040] Wilson J. M., Duncan N. M., Marin P. J., Brown L. E., Loenneke J. P., Wilson S. M. C., Jo E., Lowery R. P., Ugrinowitsch C. (2013). Meta-analysis of postactivation potentiation and power: effects of conditioning activity, volume, gender, rest peiods, and training status. Journal of Strength and Conditioning Research.

[j_hukin-2022-0068_ref_041] Yanaoka T., Iwata R., Yoshimura A., Hirose N. (2021). A 1-Minute Re-warm Up at High-Intensity Improves Sprint Performance During the Loughborough Intermittent Shuttle Test. Frontiers in Physiology.

[j_hukin-2022-0068_ref_042] Yanaoka T., Hamada Y., Fujihira K., Yamamoto R., Iwata R., Miyashita M., Hirose N. (2020). High-intensity cycling re-warm-up within a very short time-frame increases the subsequent intermittent sprint performance. European Journal of Sport Science.

[j_hukin-2022-0068_ref_043] Yanaoka T., Kashiwabara K., Masuda Y., Yamagami J., Kurata K., Takagi S., Miyashita M., Hirose N. (2018a). The effect of half-time re-warm-up duration on intermittent sprint performance. Journal of Sports Science and Medicine.

[j_hukin-2022-0068_ref_044] Yanaoka T., Hamada Y., Kashiwabara K., Kurata K., Yamamoto R., Masashi Miyashita, N. H. (2018b). Very-short-duration, low-intensity half-time re–warm-up increases subsequent intermittent sprint performance. Journal of Strength and Conditioning Research.

